# Expandable intramedullary nails in lower limb trauma: a systematic review of clinical and radiological outcomes

**DOI:** 10.1007/s11751-013-0156-9

**Published:** 2013-03-10

**Authors:** David M. Rose, Toby O. Smith, Dominic Nielsen, Caroline B. Hing

**Affiliations:** 1 St George’s Hospital, Blackshaw Road, Tooting, London, SW17 0QT UK; 2 University of East Anglia, Norwich, UK

**Keywords:** Tibial fracture, Femoral fracture, Expandable nail

## Abstract

This study systematically reviews the evidence-base for the use of expandable nails in the treatment of acute diaphyseal fractures of the lower limb. Both electronic and hand searches were undertaken of the published and grey literature to 1 December 2011. A total of 154 citations were identified, of which 15 were deemed suitable and assessed with the Critical Appraisals Skills Programme tool. A total of 625 nailing procedures were performed in 620 patients: 279 femoral and 346 tibial nails. The expandable nail was found to be significantly quicker to insert than interlocked nails (*p* < 0.05), and the total incidence of non-union or other complication was 13 and 14 % for expandable femoral and tibial nails, respectively. Notable complications with the expandable nail included fracture propagation on nail inflation in 2.5 % and post-operative shortening in 3.3 %. Device failure secondary to problems with the expansion mechanism was seen in 2.9 %. The rate of non-union and infection following expandable nailing was 3.1 and 1.4 %, respectively. Despite promising initial results, there remains a paucity of good quality studies to support the use of expandable nails over interlocked nails for the treatment of acute diaphyseal fractures of the lower limb.

## Introduction

Locked intramedullary (IM) nailing is the gold standard treatment of diaphyseal long bone fractures of the lower limb. It provides rapid fracture stabilisation using a minimally invasive approach and allows early mobilisation and return of function to the injured limb [1–4]. Expandable nails, such as the Fixion™ nail, are a relatively new technological development. These are IM nails that are implanted without the need for a guide wire or reaming, and are inflated with saline to conform anatomically to the diaphyseal cortex [5, 6]. The inflated nail abuts the endosteal surface of the bone and provides an interference fit which is theoretically stable enough to maintain fracture reduction and avoid the need for locking screws [5].

Data from a retrospective comparative study suggest that the clinical outcome of femoral diaphyseal fractures treated with an expandable nail may be superior to those treated with a standard locked nail [7]. Furthermore, as reaming and interlocking screws are not necessary with expandable nails, other potential advantages over standard locked nails have been cited as a reduction in perioperative blood loss, operative time and exposure to ionising radiation [5, 8]. In the multiply injured patient who requires stabilisation of a long bone fracture of the lower limb and in whom a rapid procedure with minimal surgical trauma is advantageous, the expandable nail is a theoretically attractive device.

In this systematic review, we set out to determine, based on current evidence, the clinical and radiological outcomes of expandable IM nails when used in the treatment of diaphyseal fractures of the femur and tibia. Our secondary aims were to compare its performance with that of the gold standard locked IM nail and to determine whether these devices have a role in the certain situations such as polytrauma.

## Materials and methods

### Systematic review

One reviewer (TS) performed a PRISMA compliant search of the electronic databases AMED, CINAHL, EMBASE and MEDLINE via the Ovid platform from inception to 1 December 2011. The Cochrane Central Register of Controlled Trials and unpublished database engines including SIGLE (System for Information on Grey Literature in Europe), the National Research Register (UK), UKCRN Portfolio Database, National Technical Information Service and the Current Controlled Trials database were then reviewed. The search strategy is presented in Tables 1 and 2. This was modified for each of the databases. The reference lists of all potentially eligible studies and corresponding authors from all included studies were contacted to identify any papers initially omitted from the electronic search.

**Table 1:** Search strategy for published databases (AMED, CINAHL, MEDLINE, EMBASE, BNI, HMIC)

Number	Term
1	Nail.ti.ab
2	Expand$ ti.ab
3	Exp.balloon
4	Fixon.ti.ab
5	Exp.fractures,bone
6	Union.ti.ab
7	Patholog$ti.ab
8	Fusion.ti.ab
9	Exp/Bone
10	Humer$. ti.ab
11	Tibia$ti.ab
12	Femoral. ti.ab
13	Exp.femur
14	Subtalar. ti.ab
15	Exp.ankle
16	OR/2–4
17	AND/1,16
18	OR/5–8
19	OR/9–15
20	AND/17–19
21	Remove duplicated/20

**Table 2:** Search strategy and results for unpublished literature databases

Database	Term	Result	Result
OpenSIGLE (System for Information on Grey Literature in Europe); WHO International Clinical Trials Registry Platform; UKCRN Portfolio Database; National Technical Information Service; Current Controlled Trials database; National Research Register	1	Expandable nail.tw.	0
	2	Orthopaedic nail.tw	0
	3	Orthopaedic nail.tw	0
	4	AND/2,3	0
	5	OR/1,4	0

Study identification was initially performed by one reviewer (TS) and subsequently verified by two reviewers (CH and DR) after consulting the titles and abstracts. We initially included all studies which presented the clinical and/or radiological results of patients treated with an expandable IM nail following an upper or lower limb fracture or had undergone prophylactic fracture fixation for insufficiency fractures in conditions such as osteoporosis or skeletal metastases. All cadaveric or animal studies and all biomechanical studies not involving living humans were excluded. We did not exclude studies based on methodological quality, language or age. Following the initial review, we included only those papers which reported the findings of patients managed with an expandable nail for acute diaphyseal lower limb (femoral or tibial) fractures. Full texts were ordered for all papers initially considered eligible and after satisfying the eligibility criteria were then included in the final review.

### Data extraction

One reviewer (DR) initially extracted the relevant data from the included studies. This was then independently verified by a second reviewer (CH). The data extracted from each study included cohort characteristics (age, gender, fracture), treatment (surgical and post-operative management), outcome measures, results and duration of follow-up. All outcome measures provided in each paper were included.

### Methodological appraisal

Studies identified in the search strategy and included in this review were randomised controlled trials (RCT), case series or case–control studies. The CASP critical appraisal tool was adopted and modified specifically to address this clinical area. Accordingly, twenty critical appraisal questions were asked of each paper. These are itemised in Table 3 and used to assess the internal and external validity of each included study. Each study was evaluated against this checklist by one reviewer (DR) and verified by a second (CH). Any disagreements were resolved by consensus.

**Table 3:** CASP results

Study	Total (20)
Lepore et al. [7]	15
Smith et al. [15]	14
Steinberg et al. [16]	14
Ben Galim et al. [9]	13
Bi et al. [6]	12
Fortis et al. [11]	10
Panidis et al. [13]	10
Bekmezci et al. [17]	9
Zocalli et al. [10]	9
Cilli et al. [19]	9
Kapoor et al. [18]	8
Capelli et al. [8]	7
Ozturk et al. [12]	6
Lepore et al. [5]	5
Pascarella et al. [14]	4

Criteria

1. Did the review ask a clearly focused question?

2. Was the population clearly defined?

3. Was a cohort study design appropriate (i.e. was one intervention reviewed or 2 or more when a RCT may have been more appropriate)?

4. Did the paper state a clear research question?

5. Was the cohort representative of this population?

6. Was everybody included who should have been included?

7. Were the appropriate outcome measurements used?

8. Did the study identify if the outcome measurements are valid and reliable for this population?

9. Were the measurement methods similar for the different groups?

10. Were the subjects/blinded to the intervention?

11. Was the assessor blinded to the intervention?

12. Did the paper control for confounding variables, for example, population heterogeneity/interventional heterogeneity?

13. Did more than 85 % of the cohort who started the study finish the study?

14. Was the follow-up period sufficiently long enough to determine clinical/radiological outcomes?

15. Has the paper clearly defined the outcomes of the study?

16. Has the paper looked at differences between populations or interventions and assess for this with appropriate statistical test?

17. Were confidence intervals presented to assess the precision of the statistical result?

18. Can the results be attributed to bias/confounding/chance event rather than the effect of the intervention specifically?

19. Are the subjects of the study reflective of this typical population?

20. Do the results of this study fit with other available evidence?

### Data analysis

An observation of the findings from the data extraction table indicated a large degree of between-study heterogeneity in respect of cohort characteristics, fracture types, interventions and outcomes recorded. Accordingly, it was deemed inappropriate to pool these results in a meta-analysis. We therefore analysed the results of the included studies as a narrative systematic review.

## Results

### Search results

**Fig. 1:**
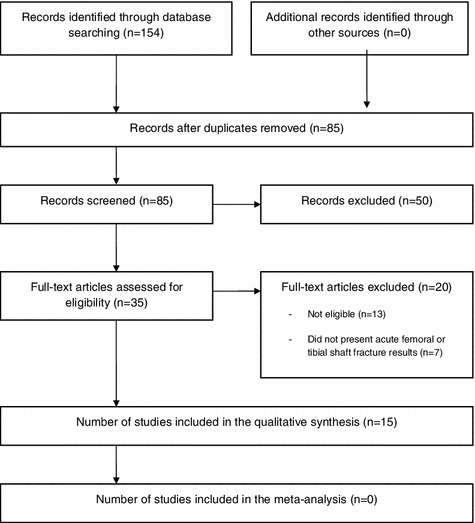
PRISMA chart showing the results of the search strategy

The results of the search strategy are presented in the PRISMA flow chart (Fig. 1). In total, 154 citations were identified from the published and unpublished literature. Of these, 15 were deemed eligible and included in the final review.

### Methodological appraisal

The appraisal indicated that the evidence-base presented with a number of methodological limitations, most strikingly the recruitment of small sample sizes with insufficient power (Tables 4, 5). A major recurrent limitation was the assessment of clinical outcomes for this patient group. None of the studies documented that the outcome measurements adopted were reliable or valid for this population (Table 5). Similarly, when analysed, none of the studies reported their results with confidence intervals. Finally, none of the studies blinded their assessors or patients to the implant used to manage their fracture. Whilst it would not have been possible to blind the surgeon to the type of nail used, the blinding of assessors or clinicians would have been feasible and could have reduced ascertainment bias. Nonetheless, a strength of the evidence-base was that in all but 4 studies, there was minimal loss of patients during the follow-up period (Table 4). Finally, whilst there were methodological flaws in many of the studies, the population recruited was clearly defined in all but six and was representative of typical acute femoral and tibial fractures in the final review.

**Table 4:** Study demographics

Study	Study design	Subjects	Mean age, years (range)	Gender (f/m)	Limbs	Follow-up (months)
Lepore et al. [7]	Case–control	86	32.5 (18–79)	22/64	86	N/S
Smith et al. [15]	Case series	48	25.2 (18–49)	15/33	49	16 (9–32)
Steinberg et al. [16]	Case series	54	40 (19–84)	17/37	54	14 (12–24)
Ben Galim et al. [9]	RCT	53	39.1 (17–84)	14/39	53	24
Bi et al. [6]	RCT	46	38.4 (20–74)	19/27	46	16 (12–34)
Fortis et al. [11]	Case series	26	38 (17–78)	5/21	26	24
Panidis et al. [13]	Case series	20	25 (18–62)	N/S	20	15
Bekmezci et al. [17]	Case series	20	31 (15–75)	10/10	20	26 (9–38)
Zocalli et al. [10]	Case–control	93	36 (17–62)	N/S	96	15
Cilli et al. [19]	Case series	20	34 (18–70)	5/15	20	10 (5–16)
Kapoor et al. [18]	Case series	32	31.8 (18–62)	8/24	32	28 (12–43)
Capelli et al. [8]	Case series	22	48 (8–68)	10/12	22	6
Ozturk et al. [12]	Case series	42	N/S	N/S	42	N/S
Lepore et al. [5]	Case series	39	N/S	N/S	39	N/S
Pascarella et al. [14]	Case series	19	37 (14–78)	6/13	20	N/S

*RCT* randomised controlled trial, *N/S* not specified, *f* female, *m* male

**Table 5:** Study characteristics

Study	Limbs		Open/closed fracture	Fracture type (AO-number)	Nail allocation	Reaming	Mean time to union, months (range)	Complications
Lepore et al. [7]	86		Closed	86 Femora (**32A/B**-86)	43 Stratec 43 Fixion	43 reamed 19 reamed	L—4 (3–9) E—3 (3–9)	L—1 nail breakage
Smith et al. [15]	49		37 closed 12 open	22 Femora (**A1**-4; **A2**-6; **A3**-7; **B1**-1; **B2**-4) 27 Tibiae (**A1**-4; **A2**-11; **A3**-9; **B2**-3)	22 Fixion 27 Fixion	46/49 reamed	F—4 (2–6) T—3 (2–6)	E—2 tibial delayed unions, 1 femoral and 1 tibial non-union, 5 tibial + 6 femoral Fx shortened, 7 propagation of Fx intra-operatively
Steinberg et al. [16]	54		27 closed 27 open	54 Tibiae (**A1**-3; **A2**-14; **A3**-16; **B1**-3; **B2**-7; **B3**-3; **C1**-4; **C2**-3; **C3**-1)	54 Fixion	30/54 reamed	3 (1–7)	E—2 superficial infections, 3 deep infections, 2 bone shortening, 1 Fx propagation, 1 distal malalignment
Ben Galim et al. [9]	53		26 closed 27 open	53 Tibiae (**A1**-10; **A2**-10; **A3**-12; **B1**-4; **B2**-8; **B3**-9)	26 Mathys 27 Fixion	7 reamed 5 reamed	L—3 E—4	L—3 neurological deficits, 3 infections E—no complications
Bi et al. [6]	46		34 closed 12 open	46 Tibiae (**42**-46)	24 Locked 22 Fixion	46 reamed	L—4 ± 1 E—3 ± 1	L—1 infection, 1 nail breakage
Fortis et al. [11]	26		20 closed 6 open	26 Tibiae (**A1**-3; **A2**-8; **A3**-8; **B1**-3; **B2**-3; **B3**-1)	26 Fixion	Unreamed	3	E—1 nail failed to expand
Panidis et al. [13]	20		N/S	11 Femora (**31**-4; **32**-6; **33**-1) 9 Tibiae (**42**-9)	20 Fixion	Unreamed	N/S	E—3 nails bent at tip, 1 nail failed to expand
Bekmezci et al. [17]	20		Closed	20 Femora (**32A/B**-20)	20 Fixion	N/S	N/S	No complications
Zocalli et al. [10]	96		95 closed 1 open	45 Femora (**A2**-27; **A3**-4; **B1**-7; **B2**-5; N/S-2) 51 Tibiae (**A2**-16; **A3**-30; **B2**-1; **B3**-4)	24 locked 21 Fixion 24 locked 27 Fixion	E—4 reamed	N/S	E—1 Fx widening, 1 pneumatic system rupture, 1 nail incarceration, 2 tibial delayed union
Cilli et al. [19]	20		19 closed 1 open	20 Femora (**32**-20)	20 Fixion	N/S	15.2 (12–24)	No complications
Kapoor et al. [18]	32		Closed	22 Femora (**A1**-3; **A2**-4; **A3**-10; **B1**-2; **B2**-2; **B3**-1) 10 Tibiae (**A1**-2; **A2**-3; **A3**-2; **B1**-1; **B2**-1; **B3**-1)	22 Fixion 10 Fixion	22 reamed 10 reamed	F—5 (4–11) T—5 (3–9)	F—2 delayed union, 1 femoral nail bent after repeated trauma T—1 delayed union with infection
Capelli et al. [8]	22		Closed	22 Tibiae (**A1**-2; **A2**-4; **A3**-16)	22 Fixion	Unreamed	T—4 (3–5)	T—1 superficial infection
Ozturk et al. [12]	42		N/S	29 Femora (**32-**29) 13 Tibiae (**42-**13)	29 Fixion 13 Fixion	N/S	N/S	E—2 rotationally unstable nails progressed to non-union, 1 nail deflation with pseudoarthrosis, 1 longitudinal fracture propagation, 1 nail bent during weight bearing with further fracture development, 1 inserter connector breakage and nail could not be inflated or removed
Lepore et al. [5]	39		N/S	9 Femora (**32A**-8; **32C2**-1) 13 Tibiae (**42A**-12; **42B**-1) (17 unavailable for follow-up)	9 Fixion 13 Fixion	16 reamed	F—2 (N/S) T—3 (N/S)	E—2 fracture propagation on nail insertion
Pascarella et al. [14]	20		N/S	8 Femora (**32-**8) 12 Tibiae (**42**-12)	8 Fixion 12 Fixion	20 reamed	F—5 (4–7) T—4 (3–6)	E—1 refracture with bending of nail, 1 nail thread bit of inflator broke off and remained in nail, 1 nail sprang a leak

*OI* osteogenesis imperfecta, *N/S* not specified, *L* locked nail, *E* expandable nail, *Fx* fracture, *F* femur, *T* tibia

Bold values indicate AO long bone fracture classification

### Study characteristics

The study characteristics of the 15 studies are summarised in Tables 4 and 5. As this illustrates, only two studies were prospective RCTs comparing the use of conventional interlocking IM nails to an expandable nail [6, 9] and two studies were case–control studies [7, 10]. The remaining 11 studies were case series evaluating the outcomes of expandable nailing for acute diaphyseal fractures of the femur and/or tibia [5, 8, 11–19]. In total, 625 IM nailing procedures were performed in 620 patients; 279 of these were femoral IM nailings, and 346 were tibial IM nailings. Of the 279 femoral IM nail procedures, 212 were performed with an expandable device, and 67 with a locked IM nail. Of the 346 tibial IM nailing procedures, 272 were performed with an expandable device and 74 with a locked IM nail.

**Fig. 2:**
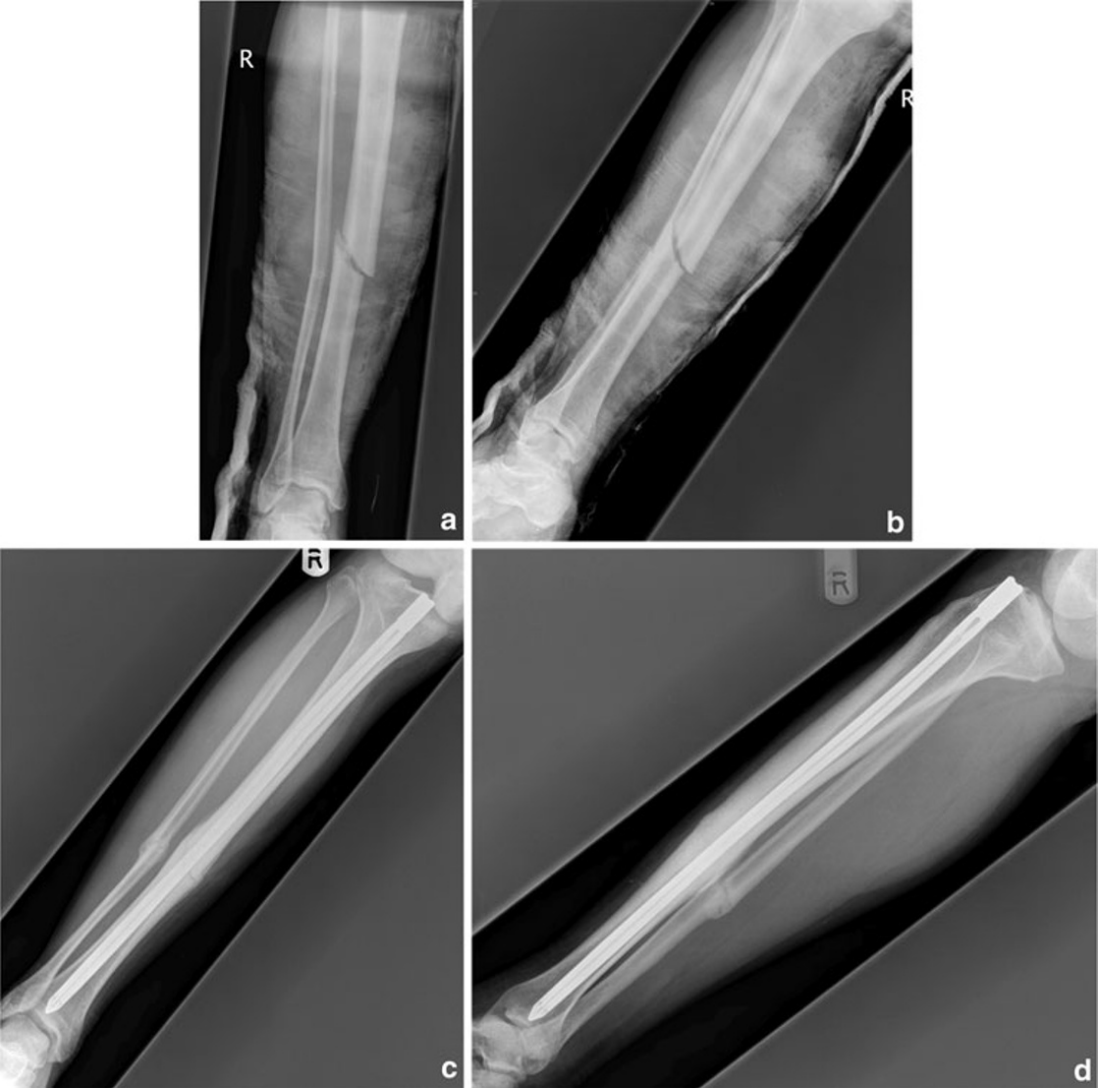
**a**–**d** Radiographs demonstrating treatment of an acute tibial shaft fracture with the Fixion™ expandable nail

The device used in all studies was the Fixion nail (Disc-O-Tech Medical Technologies Ltd, Tel Aviv, Israel) (Fig. 2). Mean follow-up periods ranged from 6 to 26 months (Table 4).

### Clinical and radiological outcomes

For the purpose of this review, we subdivided the clinical and radiological outcomes of the expandable nail according to anatomical region (femur or tibia).

### Femoral nails

#### Interlocking nails versus expandable nails

Two case–control studies compared the clinical outcome of expandable with locked IM nails in the treatment of diaphyseal femoral fractures [7, 10]. Lepore et al. [7] reported superior results in 43 patients who had undergone expandable nailing compared to a group of matched patients who had a slotted locked IM nail (Stratec, Welwyn Garden City, UK) for a closed diaphyseal femoral fracture. They found the mean time for clinical (3.8 vs. 6.8 months) and radiographic (3.5 vs. 7.5 months) union to be significantly shorter in the Fixion nail versus the Stratec nail fixation groups (*p* = 0.02; *p* = 0.01). There were also a greater number of complications in those who underwent locked IM nail fixation; 2 patients required a further operation to remove prominent hardware, and 5 others required dynamisation of the implant at 6 months due to failure to achieve union. In another patient, a locked IM nail broke resulting in further surgery to exchange the broken nail. No complications were reported with the expandable nail.

Zocalli et al. [10] reported significantly shorter operative time (55 vs. 74 min, *p* < 0.01) with 21 acute femoral fractures and 27 tibial fractures treated with an expandable nail, when compared to a matched control group treated with a locked IM nail. There were no other significant differences in outcome between the groups. Of note, they reported 2 cases of post-operative fracture shortening in the expandable nail cohort, as well as 1 case of intra-operative fracture widening.

These studies had limitations in their methodology. Firstly, neither study performed a power analysis to determine the number of patients necessary to show a difference between the groups. Secondly, bias may have been introduced when matching the groups as neither surgeon nor patients were blinded to the treatment modality. Thirdly, outcomes were not assessed independently with the assessor blinded to the original treatment. Finally, with reference to Lepore et al.’s study, the exact point of clinical and radiographic fracture union is clearly difficult to ascertain with any reliability or reproducibility, particularly since patient follow-up was on a 2 monthly basis.

#### Case series results of expandable nail in femoral fracture fixation

There were results of eight case series of acute femoral fractures treated with expandable nails [5, 12–15, 17–19]. In general, there were severe methodological limitations to these papers, with small numbers of patients and the lack of blinding or independent assessment of outcomes. In addition, Lepore et al.’s study had a poor follow-up rate, with only 29 of the original cohort of 41 patients available for follow-up [5].

#### Healing time-frame

Three studies reported time to radiological union following femoral fracture fixation with an expandable nail [5, 15, 18]. These studies demonstrated radiological fracture union at a mean of 13 weeks, ranging from 9.5 to 16 weeks [5, 18]. Kapoor et al. [18] also presented their duration until clinical union, reporting this to be 11.5 (range 8–28) weeks.

#### Operative and fluoroscopy time

The duration of surgical procedure was reported in three studies to have an overall mean duration of 67 (range 43.4–90) min [13, 15, 18].

Two studies presented their duration of fluoroscopy use to be an overall mean of 56 (range 28–84) s [13, 18].

#### Complications (Table 6)

Non-union or delayed union resulted from 5 of 212 (2.4 %) femoral expandable nailing procedures. In total, three studies reported femoral non-union or delayed union [14, 15, 18]. All patients required further intervention to achieve union.

**Table 6:** Complications of femoral and tibial expandable nails

Complication	Overall complication rate (%)	Femoral	Tibial	References
Non-union or delayed union	3.1	2.4 %	3.7 %	[10, 12–16, 18]
Shortening	3.3	3.8 %	2.9 %	[10, 15, 16]
Fracture propagation	2.5	N/A	N/A	[5, 10, 12, 15, 16]
Implant failure	2.9	4.2 %	1.8 %	[6, 10–14, 18]
Infection	1.4	0 %	2.6 %	[8, 16, 18]

*N/A* data not available

One case of intra-operative extension of the fracture was reported within the literature. Ozturk et al. [12] reported that in one patient with osteogenesis imperfecta, a new longitudinal fracture occurred during inflation of the femoral nail. Conversion to a conventional locked nail, supplemented with cerclage wire was then carried out.

Smith et al. [15] reported post-operative fracture shortening of greater than 1 cm in 6 cases with a mean shortening of 2.2 cm (range 1.1–3 cm). Each case was revised to a conventional interlocking nail. Zocalli et al. [10] also reported 2 cases of shortening of greater than 2 cm in their cohort.

Implant failure was recorded in Pascarella et al.’s [14] paper, documenting 1 case where the threaded part for the inflator broke off but remained in the nail and another case where the expandable nail leaked so the implant could not be inflated, requiring a substitute nail be used. One case of re-fracture was acknowledged by Pascarella et al. [14] in a drug abuser 2 months following the initial fracture, necessitating removal of the expandable device and revision with a conventional locked nail. Panidis [13] and Zocalli [10] reported a total of 5 cases where the expandable nail either bent or failed in some way during the insertion process, also requiring exchange nailing. In Kapoor et al.’s [18] series, an expandable nail bent in the post-operative period but the resultant deformity was accepted and the femur went onto mal-union.

### Tibial nails

#### Interlocking nails versus expandable nails

Two RCTs [6, 9] and 1 case–control study [10] compared expandable and locking IM nail fixation for acute diaphyseal fractures of the tibia. Zocalli reported that operative times for insertion of the expandable nail for tibial fractures were significantly shorter (*p* < 0.01) than the interlocking nail [10]. Anecdotally, the authors noted that those patients who had undergone expandable nail fixation appeared to heal slower that those who had undergone conventional locked nailing but did not provide clear data on fracture union times in order to support their opinion. They observed a single case of post-operative shortening with the expandable nail and two cases of delayed union at 6 months requiring further surgery.

Results favouring expandable nail fixation were also reported by Bi et al. [6]. They observed those patients treated with the device had a significant reduction in operative time, intra-operative blood loss, exposure to ionising radiation, length of hospital stay and time to fracture union when compared to those managed with a conventional interlocking nail (*p* < 0.05). However, no significant differences were reported with respect to clinical outcome as measured by the Johner–Wruhs scoring system [20], and complication rates were lower in patients who underwent locking IM nail fixation. In the expandable nail cohort, there was one non-union, one delayed union requiring dynamic ring fixation and one nail breakage. There were no complications noted in the interlocking nail group, but, due to the small numbers, this difference was not significant (*p* > 0.05).

The duration of surgery was significantly shorter for patients who underwent expandable nailing compared to conventional locked nail fixation in Ben-Galim’s study (*p* < 0.001) [9]. They reported those patients who underwent conventional locked nail fixation experienced a significantly greater incidence of re-hospitalisation, re-operation (*p* < 0.0001) and, more frequently, required removal of the nail (*p* = 0.01) as compared to those in the expandable nail group. In addition, there was a non-significant trend towards a higher rate of peroneal nerve palsy and infection in the locked nail group; three cases were reported for each of these complications in the interlocking nail groups, whilst none in the expandable nail group. There was also a trend towards a reduced time to fracture union for the expandable nail group (11.5 vs. 17 weeks), although not statistically significant (*p* > 0.05).

The quality of these studies was undermined by the absence of power analyses, blinding or independent assessment of outcomes as well as low numbers in the respective treatment groups.

#### Case series results of expandable nail for tibial fracture fixation

We report the results of 9 case series of acute tibial fractures treated with expandable nails [5, 8, 11–16, 18]. As with previous papers, there are several methodological limitations to these papers with small numbers of patients and the lack of blinding or independent assessment of outcomes.

#### Operative and fluoroscopy time

The duration of the surgical procedure was assessed in six studies [11, 13–16, 18]. These indicated a total mean operative time of 48.3 (35–84) min. Steinberg et al. [16] also compared operative duration of reamed and unreamed expandable nails; unreamed nailing procedures were significantly faster, with a mean time of 56 (30–80) min, whilst reamed nails took a mean time of 103 (range 40–185) min, *p* < 0.0001. They also reported a significantly shorter operative time with 8.5 mm vs. 10 mm expandable nails; 70 (30–180) min versus 103 (55–185) min, respectively (*p* = 0.005). However, there was no significant difference in duration of surgical procedure for closed versus open tibial fractures managed with an expandable nail; 88 (40–185) min versus 78 (30–180) min, respectively (*p* = 0.43).

Three studies reported a mean duration of 27 s (10–54) of fluoroscopic exposure during the surgical procedure [11, 13, 18].

### Hospital length of stay

One study assessed the length of stay for their patients following expandable nail fixation for tibial fractures. Steinberg et al. [16] reported that mean hospital duration in their cohort of 54 acute midshaft tibial fractures was 15 (range 3–102) days.

#### Fracture time-frames

Time to union was reported in 6 studies [8, 11, 14–16, 18]. They reported an overall mean duration of 13.7 (range 10.3–16) weeks.

#### Functional outcomes

Three studies assessed functional outcome following expandable nailing of tibial fractures. Fortis et al. [11] assessed the Iowa Knee and Ankle Score at 2 years, reporting a mean score of 93 and 95, respectively. Subjective clinical scores were obtained in Capelli’s [8] study. In their cohort of 19 tibial patients, they reported clinical results to be excellent in 16 patients and good in three. Pascarella et al. [14] assessed the duration until weight bearing; mean time until partial and total weight bearing were recorded as 7 and 40 days, respectively.

#### Complications (Table 6)

Non-union or delayed union resulted from 10 of 272 (3.7 %) tibial expandable nailing procedures. In total, 5 case series reported tibial non-union or delayed union [12, 13, 15, 16, 18]. Re-operation was required in all but 1 of these 10 patients.

Rotational instability following implantation was noted by Fortis et al. [11] and Ozturk et al. [12], resulting in tibial mal- and non-union, respectively. This was due to implant failure in the latter study.

Smith et al. [15] reported 5 cases of acute post-operative fracture shortening of >1 cm with mean shortening of 2.1 cm, ranging from 1.5 to 2.5 cm. In each case of shortening, re-operation with conversion to a conventional locked nail was carried out. In Steinberg et al.’s series, two cases of fracture shortening of >1 cm were noted, resulting in proximal protrusion of the expandable nail into the knee joint [16].

Steinberg et al. [16] also documented a single case of intra-operative extension of a tibial shaft fracture on expansion of an expandable nail, converting an A2 to a C2 fracture pattern. Eleven further cases of intra-operative fracture extension with the expandable nail were reported in 3 other studies, but the authors did not specifically detail whether they occurred in the femur or the tibia [5, 10, 15].

Seven cases of infection following tibial expandable nailing were reported; 5 of these came from a single [16] series, and 2 from separate series [8, 18]. All required surgical debridement.

Four cases of implant failure were reported. Fortis et al. [11] reported one defective valve leading to a nail not inflating. Two cases of implant failure were presented in Ozturk’s [12] series: in the first of these, the expandable nail bent once weight bearing was commenced and revision to a conventional locked intramedullary nail was then carried out; in the second case, the nail was damaged during the process of insertion meaning that it could neither be inflated nor removed; non-union subsequently developed which required revision of the nail and bone autograft. In a similar case, Panidis et al. [13] reported one case of tibial fracture in which a nail failed to expand and was left un-inflated; as a consequence, the fracture went onto non-union.

Two patients in the Fortis et al. [11] cohort of 26 tibial fractures developed anterior knee pain following expandable nail fixation; neither patient wished to have the nail removed.

Finally, one patient in Steinberg et al.’s [16] cohort developed compartment syndrome in the early post-operative period and required fasciotomy.

## Discussion

Historical evidence suggests that the best treatment for diaphyseal fractures of the lower limb is locked IM nailing [1–4]. In this systematic review, we sought to determine whether the expandable nail offers the trauma surgeon an acceptable and safe alternative to the locked IM nail. Certainly, these data suggest the expandable nail system appears to be significantly quicker to implant than a standard locked IM nail [6, 9, 10]. The reason for this is presumably that the nail does not always require reaming prior to insertion and never requires locking screws, which means that some potentially lengthy steps of the nailing procedure are avoided. It follows that in the multiply injured patient, where rapid surgical procedures that result in minimal systemic insult may be beneficial, the expandable nail is a potentially useful device.

Pooled RCT data from studies involving reamed IM nailing of diaphyseal femoral [21–24] and tibial [25–28] fractures demonstrate a non-/delayed union rate of 5 % for both anatomical regions. This rises to 11 % (tibial fractures) and 24 % (femoral fractures) should nails be inserted unreamed [21–28]. The results of the present study show that the expandable nail compares favourably with the locked IM nail with respect to fracture union rates, with a non-/delayed union rate of 2.4 and 3.7 % when used in the femur and tibia, respectively.

The overall complication rate for expandable nailing was 13 % for femoral nails and 14 % for tibial nails, whilst the rate of re-operation was 10 and 11 %, respectively. Data from the SPRINT trial indicate a re-operation rate also of 11 % for reamed interlocking nails in acute tibial shaft fractures [29]. Beazley et al. [30] recently reviewed the use of expandable nails in the treatment of acute tibial shaft fractures alone. The present study has demonstrated a similar complication rate when the expandable nail is used for acute femoral fractures.

Although this systematic review demonstrates that the initial results from the use of expandable nails are promising, we would caution that most of the studies involved demonstrated numerous methodological weaknesses. There were 4 comparative studies; 2 of which were RCTs [6, 9], and 2 case–controls [7, 10]. The other 11 studies were case series. All 15 studies had small cohorts of patients; outcome measures were, in general, poorly defined and suffered from a lack of independent assessment. Several studies measured time to clinical and radiological fracture union; this outcome measure is clearly open to inaccuracy given the often-lengthy intervals between fracture clinic appointments, as well as the difficulty of determining exactly when a fracture has united.

One of the main purported advantages of the expandable nail is that it does not require reaming of the intramedullary canal during insertion. Reaming allows insertion of larger nails, thereby improving construct stability, reducing time to fracture union and the rate of hardware failure [31]. However, controversy regarding the use of reaming, particularly in those patients with multiple injuries, remains. Although rises in intramedullary pressure with subsequent intravasation of intramedullary debris have been shown to be associated with both reamed and unreamed nail insertion [32, 33], this effect appears to be particularly severe with reaming [34, 35]. Microscopic pulmonary emboli may result in a reduction in pulmonary function and the development of acute respiratory distress syndrome (ARDS), particularly in the multiply injured patient [35–37]. This has led some authors to favour a “damage control” approach in this severely injured subset of trauma patients with long bone fractures [38, 39]. Of the studies in this review which described whether reaming had been performed, 35 % of femora and 38 % of tibiae had been reamed, suggesting that the theoretical advantage of avoidance of reaming with the expandable nail is not always borne out in practice.

At present, the indications for expandable nail fixation in the lower limb appear to be broad, with the manufacturer claiming that any diaphyseal fracture greater than 5 cm from either proximal or distal metaphyseal regions may be treated with the device. Biomechanical data suggest that expandable nails may be more suitable for use with specific fracture patterns; Maher et al. [40] compared the Fixion nail with a standard locked nail in a tibial fracture model, finding that spiral fracture patterns, rather than transverse fractures, were more suitable for expandable nail fixation. However, construct bending and torsional stiffness, rather than resistance to axial loading and therefore potential for fracture shortening, were tested. Further relevant studies would be helpful in order to clarify those types of fractures best indicated for expandable nail fixation, as opposed to those that would be more suitable for other interventions.

Important complications associated with the expandable nail are post-operative shortening and fracture propagation on inflation of the nail. Three studies reported post-operative shortening [10, 15, 16], and a total of 3.3 % of all limbs implanted with the expandable nail demonstrated this complication at follow-up. In addition, 2.5 % of limbs underwent fracture propagation on inflation of the expandable nail [5, 10, 12, 15, 16]. Smith et al. [15] noted a total of five tibial and six femoral fractures in which the treated bone had become shortened by greater than 1 cm by the 6 week post-operative examination. In fact, this single study accounted for 69 % of all cases of fracture shortening with the expandable nail reported in the literature. They postulated that this was due to fracture propagation during inflation of the nail, indicating that some length-stable fractures had become unstable following implantation. As a consequence, their prospective cohort study was terminated early due to the unacceptably high complication rate [15]. In order to achieve a tight interference fit that is axially and rotationally stable without the need for locking screws, the nail needs to be inflated to a maximum of 70 atmospheres [5]. There is an appreciable risk of propagating any occult fracture lines which may be initially undetectable on plain radiographs during the inflation process. Since most of the literature detailed in this systematic review did not specifically measure post-operative leg lengths, we suspect that the true rate of post-operative shortening as a result of fracture propagation and axial instability may be higher than is reported in the present data.

Removal of a bent expandable femoral nail has been reported in the literature by Bek et al. [41]. In their case report, a 32° bent nail in a re-fractured femur was initially straightened to decrease the angle to 10° before the fracture site was drilled. One of the four metal bars of the nail was then cut to allow complete straightening of the nail and removal. In our systematic review, three papers reported cases of nails bending with further fracture both in the femur and in the tibia [12, 14, 18] which is a potential concern. In Kapoor et al.’s series [18], a bent femoral Fixion nail was left in situ, the femur eventually uniting in a shortened and angulated position. Pascarella et al. [14] and Ozturk et al. [12] observed bending of the Fixion nail in the femur and tibia, respectively, both carrying out revision to a conventional locked nail following removal of the damaged device. They unfortunately did not comment on any difficulties associated with removal of the bent nails.

The expandable nail is also used in the treatment of humeral fractures, and several authors have described cases of failure of the device leading to deflation within the humerus and proximal migration or failure to maintain reduction, resulting in non-union [12, 42]. In the present study of expandable nail usage in the lower limb, the device failure rate was 2.9 %, which is similar to the failure rate of the device in the upper limb (3.9 %) [43]. Device failures usually result in exchange of the implant if noticed intra-operatively, or in shortening, mal- or non-union if occurring in the post-operative period. At the very least further expense in replacing the faulty device is entailed; the worst-case scenario involves revision surgery with all its attendant risks for the patient. There is also an appreciable rate of implant failure with locked nailing, however, with an auto-dynamisation rate of 5 % noted in a recent multi-centre RCT [29].

Finally, two case reports in the literature have highlighted the potential dangers of exploding expandable nails during the cremation process [44, 45]. With the increased use of expandable nails to treat fragility fractures, care should be taken to remove or decompress the nail prior to cremation.

## Conclusions

Initial data suggest that the expandable nail may be a useful device in certain situations where time factors are critical, such as in the poly-trauma patient. However, complications such as device failure and limb shortening have been reported in the present literature, and further prospective comparative studies of higher quality are required to justify its routine use in preference to the standard locked intramedullary nail.
